# Blocking Thrombin Significantly Ameliorates Experimental Autoimmune Neuritis

**DOI:** 10.3389/fneur.2018.01139

**Published:** 2019-01-04

**Authors:** Efrat Shavit-Stein, Ramona Aronovich, Constantin Sylantiev, Orna Gera, Shany G. Gofrit, Joab Chapman, Amir Dori

**Affiliations:** ^1^Department of Neurology, The Chaim Sheba Medical Center, Ramat Gan, Israel; ^2^Department of Physiology and Pharmacology, Sackler Faculty of Medicine, Tel Aviv University, Tel Aviv, Israel; ^3^Joseph Sagol Neuroscience Center, Sheba Medical Center, Ramat Gan, Israel; ^4^Department of Neurology, Sackler School of Medicine, Tel Aviv University, Tel Aviv, Israel; ^5^Robert and Martha Harden Chair in Mental and Neurological Diseases, Sackler Faculty of Medicine, Tel Aviv, Israel

**Keywords:** thrombin, experimental autoimmune neuritis, node of Ranvier, PAR1, Guillain-Barré syndrome

## Abstract

Thrombin and its protease-activated receptor 1 (PAR1) are potentially important in peripheral nerve inflammatory diseases. We studied the role of thrombin and PAR1 in rat experimental autoimmune neuritis (EAN), a model of the human Guillain-Barré syndrome (GBS). EAN was induced by bovine peripheral myelin with complete Freund's adjuvant (CFA). Thrombin activity in the sciatic nerves, clinical scores and rotarod performance were measured. Thrombin activity in the sciatic nerve was elevated in EAN compared to CFA control rats (sham rats) (*p* ≤ 0.004). The effect of blocking the thrombin-PAR1 pathway was studied using the non-selective thrombin inhibitor N-Tosyl-Lys-chloromethylketone (TLCK), and the highly specific thrombin inhibitor N-alpha 2 naphtalenesulfonylglycyl 4 amidino-phenylalaninepiperidide (NAPAP). *In-vitro* TLCK and NAPAP significantly inhibited specific thrombin activity in EAN rats sciatics (p<0.0001 for both inhibitors). Treatment with TLCK 4.4 mg/kg and NAPAP 69.8 mg/kg significantly improved clinical and rotarod scores starting at day 12 and 13 post immunization (DPI12, DPI13) respectively (*p* < 0.0001) compared to the untreated EAN rats. In nerve conduction studies, distal amplitude was significantly lower in EAN compared to sham rats (0.76 ± 0.34 vs. 9.8 ± 1.2, mV, *p* < 0.0001). Nerve conduction velocity was impaired in EAN rats (23.6 ± 2.6 vs. sham 43 ± 4.5, m/s *p* = 0.01) and was normalized by TLCK (41.2 ± 7.6 m/s, *p* < 0.05). PAR1 histology of the sciatic node of Ranvier indicated significant structural damage in the EAN rats which was prevented by TLCK treatment. These results suggest the thrombin-PAR1 pathway as a possible target for future intervention in GBS.

## Introduction

The inflammatory neuropathies are a diverse group of conditions. They share common features of inflammatory damage to myelin/axons (1, 2). Guillain-Barré syndrome (GBS) is a representative of the inflammatory neuropathies. The most common variant of GBS is acute inflammatory demyelinating polyneuropathy (AIDP), characterized by a progressive and sometimes painful weakness of limbs (3). GBS has an incidence rate of 0.8–1.9 cases per 100,000 people per year (4), and is potentially fatal. Although patients with GBS show a high recovery rate, it still causes extensive disability. 20 to 30% of the patients suffer respiratory failure (5). During the 6 months period after the onset of the disease, 20% of adult patients are still unable to walk without support (6).

The mechanisms by which nerve conduction dysfunction is generated in GBS are a topic of debate (2, 7). GBS pathophysiology includes a wide variety of immune responses. T cells are thought to participate mainly in the induction phase of the disease, whereas the progressive phase of the disease includes humoral mediated response (5). Perivascular presence of T cells can be seen in the experimental autoimmune neuritis (EAN) rat model for GBS soon after disease induction. The T cells activate monocytes to macrophages. The macrophages in turn, participate in nerve damage via inflammatory cytokines (8). B cells are thought to play a role via the creation of autoantibodies cross reactive to the lipopolysaccharide (LPS) of Campylobacter jejuni and the ganglioside GM1 (9). These autoantibodies bind nodal membrane and fix complement. They are suspected to be the cause of nodal dysfunction. They may also lead to axonal degeneration (10), and cause direct axonal damage (11). Recent evidence connects Zika infections with GBS as well, with negative anti-ganglioside antibodies (12, 13).

Current treatments of GBS include plasmapheresis and intravenous immunoglobulin (IVIG). The physiological mechanisms by which these treatments improve patients' condition are still unclear as is GBS pathophysiology itself. Despite treatment, 9–17% of GBS patients die or remain severely disabled (14). Other treatments, including adding steroids to IVIG, showed only minor short term improvement (15).

Inflammatory states are tightly related to the coagulation system, with extensive crosstalk between the two, as can be seen in the pathogenesis of vascular diseases (16, 17). This relationship was demonstrated in experimental autoimmune encephalomyelitis (EAE), the animal model for multiple sclerosis, where elevation of thrombin was shown to precede the appearance of clinical signs (18–20).

Thrombin is a key factor in the coagulation cascade and is known to participate in a wide variety of cellular and physiological processes including inflammation (21, 22), neurotrauma (23, 24), neuronal plasticity after vascular damage (25) and neuronal degeneration (26). Thrombin has a known role in central nervous system inflammatory diseases as shown in the EAE model (18, 19). Thrombin has been previously shown to have a role in the peripheral nervous system (PNS) and in pathophysiology of pain (27). We have demonstrated that the thrombin receptor protease active receptor 1 (PAR1) is localized to the nodes of Ranvier in peripheral nerves and that its activation creates a conduction block (28). These findings have suggested that excess levels of thrombin with PAR1 activation may play an important role in causing conduction failure in inflammatory diseases and thrombotic nerve diseases such as infarctions or trauma.

The aim of this study was to evaluate the effect of thrombin inhibition on clinical, histological and electrophysiological EAN parameters.

## Materials and Methods

### Animals

Females Lewis rats were purchased from Harlan (Jerusalem Israel) at age of 8 weeks, and were housed at the animal facility at Tel Aviv University Medical School 1 week prior to experiment for acclimatization. The animals were kept under standard conditions of 23 ± 1°C with a 12 h light-dark cycle and access to food and water ad libitum. The animals were in weight range of 160–180gr at the beginning of the experiment.

All experiments were approved by the animal welfare committee (M-06-004) and appropriate measures to avert pain and suffering to the rats were taken.

### Induction of EAN

The animals were immunized with 200 μl of inoculum containing 10 mg of bovine peripheral myelin (BPM), prepared in our laboratory from a bovine spinal cord roots, using the method of Kadlubowski (29), and 4 mg of *Mycobacterium tuberculosis* (strain H37RA; Difco, Detroit, Michigan) emulsified in 100 μl saline and 100 μl of complete Freund's adjuvant (CFA), injected into hind footpad and one subcutaneous site (flank). Control animals (sham) were injected with an inoculums containing *Mycobacterium tuberculosis* emulsified in saline and CFA. Immunization of the animals was marked as day 0, and the days that followed were marked as days post-immunization (DPI). Subjective clinical evaluation for neurological signs was performed before immunization and every 1–2 days (total of 24 time points) by an evaluator blinded to treatment. Severity of weakness was graded as follows: 0-undetected; 1-limp tail; 2-abnormal gait; 3-mild paraparesis; 4-severe paraparesis; 5-paraplegia; 6-paraplegia with forelimb involvement; 7-paraplegia with forelimb involvement and respiratory distress; and 8-moribund or dead (30).

### Motor Performance

Motor performance was assessed by means of a rotarod test. The rats were pre-trained before immunization to run on the rod, which rotated at a fixed speed of 13 rounds per minute. After immunization the rats were assessed every 1–2 days (total of 24 time points). The rats were allowed to run for up to 60 sec on each trial, or until they fell off. The mean of the three consecutive trials was recorded for each rat.

### Treatment Protocol for TLCK and NAPAP

N-alpha 2 naphtalenesulfonylglycyl 4 amidino-phenylalaninepiperidide (NAPAP-pefablock 76308, Fluka), N-Tosyl-Lyschloromethylketone (TLCK-616382) were purchased from Calbiochem, La Jolla, California.

The effect of treatment timing was determined based upon a preliminary study comparing 3 different treatment regimens for TLCK and NAPAP to untreated, adjuvant injected (marked sham) group and saline treated EAN (marked EAN). The specific time frames and number of animals used for the different protocols were chosen based on a previous work (31). First, an evaluation of clinical scores and rotarod scores was made in EAN versus sham rats. Follow-up was conducted in two independent repeated experiments for the sham rats (a total number of 9 animals) and four independent repeated experiments for the EAN rats (a total of 24 animals).

Second, three different protocols were compared to untreated EAN. The protocols included “early protocol” (EP) with daily injections at DPI5-15, “late protocol” (LP) with daily injections at DPI10-20, and “short protocol” (SP) with daily injections for DPI10-15. EP was started before the appearance of EAN symptoms as previously described in the literature (31, 32).

The EP experiment included 8 animals in each treatment group (NAPAP and TLCK). The SP experiment included 4 animals in each treatment group (NAPAP and TLCK). Main study was based on the preliminary study results. The LP protocol was chosen for the main *in-vivo* study, and therefore, was repeated in four independent experiments (a total number of 16 animals in the NAPAP group and 18 animals in the TLCK groups). The animals in the treatment groups were injected with 1 ml carrier solution (saline) containing either 4.4 mg/kg of TLCK (33) or 69.8 mg/kg of NAPAP intraperitoneally (IP) once daily.

### Thrombin Activity

In order to measure thrombin activity in the sciatic nerve, it was dissected, washed with ice-cold phosphate buffered saline (PBS) to remove overt blood and moved to a PBS buffer. The activity was measured as previously described in detail (34, 35). Briefly, the PBS surrounding the nerve was evaluated utilizing a fluorometric assay, quantifying the cleavage of the synthetic peptide substrate N-p-Tos-Gly-L-Pro-L-Arg-7- amido-4-methylcoumarin (tos-GPR-AMC; excitation 350 nm, emission 430 nm, Sigma T-0273). Substrate (20 μl, final concentration 10 μM) was added to 140 μl of PBS containing 0.1% bovine serum albumin (BSA). The fluorescence was measured continuously for 20 min at 37°C. The hydrolysis of tos-GPR-AMC substrate was determined from the increase in fluorescence (measured on FL-600 microplate fluorescence reader, BIOTEK, excitation wavelength/bandwidth 360/20 nm; emission wavelength/bandwidth 465/20 nm). Known concentrations of purified human thrombin (Sigma T 0553; 300 units/mg protein) were used in the same assay for calibration.

### Localization of PAR1 in Sciatic Nerve Slices

Sciatic nerves were dissected from rats at DPI32. Four animals were randomly selected from each group (sham, EAN, EAN treated with NAPAP and EAN treated with TLCK). 7–8 nodes-containing-fields were analyzed from each animal, adding up to a total of 30 per group. The sciatic nerves were fixed overnight in 4% PFA in 0.1 M phosphate buffer, pH 7.4, and then placed in 30% sucrose for 4 h. Frozen sagittal sections (50 μm) were then cut on a sliding cryostat and collected serially. Slides were then washed with PBS, blocked for 1 h in PBS containing 1% goat serum, 0.2% glycine and 0.1% Triton X-100, and incubated overnight with rabbit anti-PAR1 antibody (1:50) and mouse monoclonal anti-Caspr I (1:500; a generous gift of Professor Elior Peles). After subsequent washes in PBS, the slides were reacted with RRX- and Cy2-coupled secondary antibodies: Rhodamine Red-X-conjugated donkey anti-rabbit (1:400; Jackson Laboratories, Bar Harbor, ME, United States) and cy2-conjugated donkey anti-mouse (1:400; Jackson Laboratories).

Immunofluorescence slides were viewed and analyzed using a confocal ZEISS CLSM 410 microscope, using magnification of X630. Specific localization of PAR1 in sections from sciatic nerves was made using immunofluorescence, utilizing double staining for PAR1 together with specific markers of the node of Ranvier, including the paranodal axonal marker Caspr I. Normal node architecture was defined as the presence of Caspr paranodal staining, and PAR1 staining in the node between the paranodal Caspr stains (28). The data was evaluated blindly.

### Electrophysiological Studies

Electrophysiological tests were performed on DPI26. The rats were anesthetized with Equitezin (4% chloral hydrate, 6% sodium pentobarbital, IP, 0.5 ml/100 g). Body temperature was maintained warm by placing the rats on a heating mat. Temperature differences were minimized by conducting the study as soon as the anesthesia had taken effect and by warming the tail with a heating lamp. Electrophysiological studies were conducted on the rats tails in order to minimize an effect of local inflammation in sciatic nerve of the EAN rats (36, 37). Furthermore, length measurements were more accurate at the tail. The tail skin was cleaned carefully with alcohol before the electrodes were placed. Recordings of responses were from the muscles of the tail using a pair of ring electrodes coated with electrode jelly and placed 50 mm distal to the base of the tail. A pair of monopolar needle electrodes were inserted to a depth of 4–5 mm to stimulate the tail nerves. Stimulation was performed 10 (distal site) and 50 (proximal site) mm proximal to the recording electrodes. A ground electrode was placed between the stimulating electrode and recording electrodes. Supra-maximal stimulation, at a range of 3–5 mA was employed, and the low and high frequency filters were set at 10 Hz and 10 kHz, respectively. The responses were displayed on a fully digital recording Keypoint apparatus (Dantec, Skovlunde, Denmark). Both proximal and distal latencies were measured using time intervals from the stimulus artifact to the first deflection from the baseline. To calculate the motor nerve conduction velocity (MNCV), the distance between the stimulation sites (40 mm) was divided by the latency difference. Amplitudes of the compound muscle action potential (CMAP) from both proximal and distal stimulations were measured from the preceding baseline to negative peak.

### Splenocytes Proliferation Assay

Single-cell suspensions of freshly dissected spleens were obtained on DPI14 (*n* = 4 per each group) and assayed *ex-vivo* for their response to antigen (myelin) and immune-inducer (LPS) by a proliferation assay. Each well was seeded 2 × 10^4^ cells in 0.2 ml of proliferation medium (RPMI 1640 medium containing 10% heat-inactivated fetal calf serum, 2 mM l-glutamine, 100 U/ml penicillin, and 100 μg/ml streptomycin) containing myelin (10 μg/ml) or LPS (1 μg/ml). The experiments were performed in triplicate in 96-well, flat bottom microplates (Costar, Cambridge, USA). Cultures were incubated for 72 h in a humidified atmosphere of 95% air and 5% CO_2_ at 37°C. Cell proliferation was determined by a Colorimetric Bromodeoxyuridine (BrdU) Cell Proliferation kit (Roche Applied Science) following manufacturer's instructions. Briefly, 20 μl of BrdU labeling solution diluted 1:100 in culture medium was added to each well for the last 18 h of the assay. After removing the medium, cells were fixed, and anti-BrdU-peroxidase solution was added to each well. Cell cultures were incubated for 90 min at room temperature. The wells were washed and substrate solution was added. The absorbance was measured by means of microplate reader at 370 nm, using 492 nm as reference for subtraction.

### Statistics

Statistical analysis of the differences between clinical scores and rotarod progression were assessed by analysis of variance (ANOVA) with repeated measures. The scores at disease peak were compared by one-way ANOVA. The use of ANOVA test with Tukey's *post-hoc* analysis for clinical scores was possible due to the high number of animals, and after normal distribution was evaluated (by D'Agostino-Pearson normality test). The difference in thrombin activity was calculated using two-way ANOVA. One-way ANOVA was used to compare the means of TLCK, NAPAP and sham in the immune biochemical and electrophysiological measures. Dunnett's test was used in ANOVA with multiple comparisons. A two-way ANOVA with Sidak test for multiple comparisons was performed for the effect of EAN and progression in time on clinical score and rotarod. Comparison of immune-histochemical images was performed using Fisher's exact test. Calculations were performed using GraphPad Prism 6.

## Results

### Clinical Course and Thrombin Activity in the Sciatic Nerve of EAN

**Figure 1:**
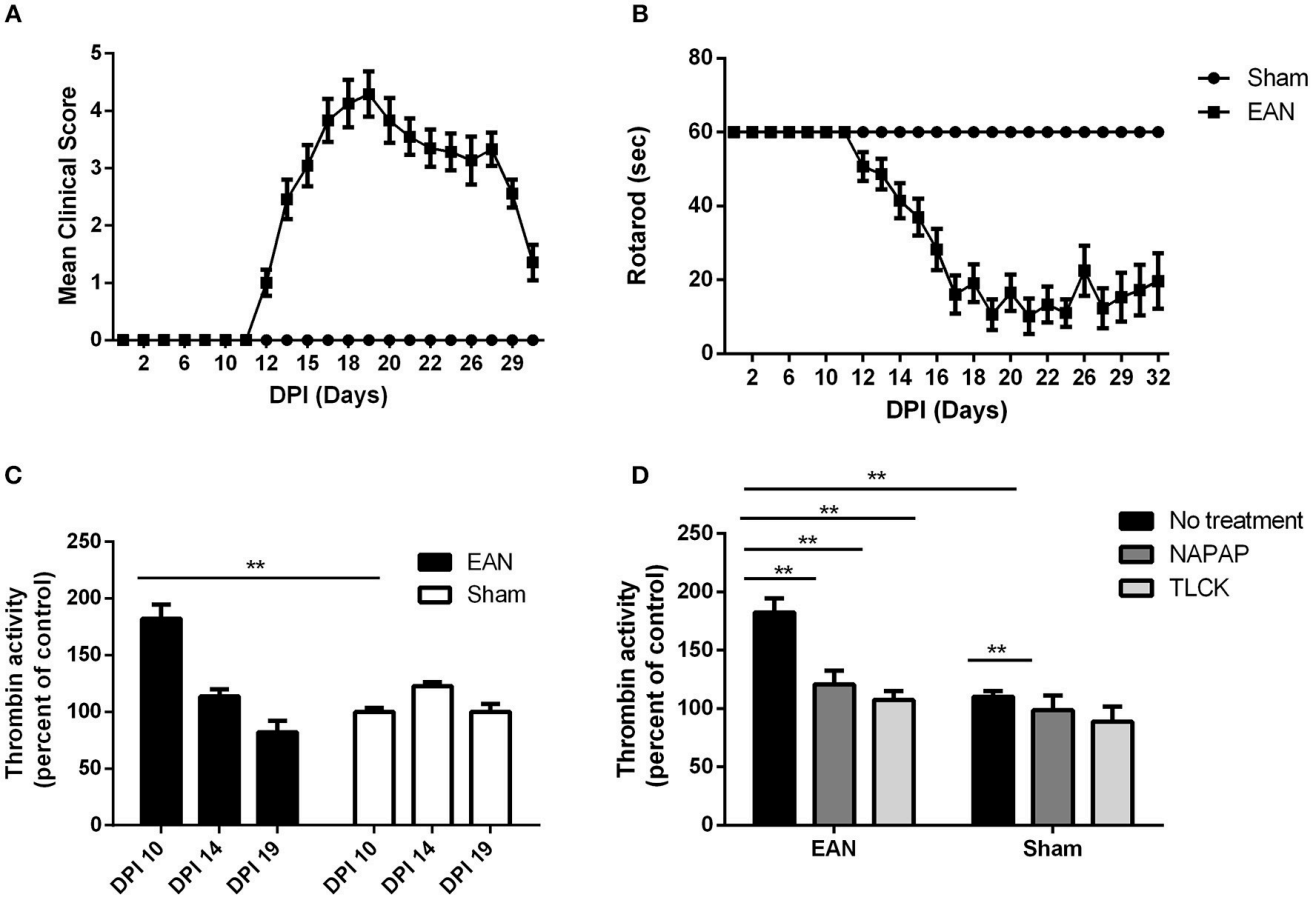
Clinical parameters and thrombin activity. Clinical parameters were followed using clinical score and rotarod score. Thrombin activity was measured using fluorometric assay, quantifying the cleavage of the synthetic peptide substrate, after the removal of the nerve from the animals. Thrombin activity following NAPAP and TLCK administration was measured *in-vitro*. **(A)** Mean clinical score (CS) of EAN rats compare to sham. **(B)** Rotaroad score (RR) of EAN rats compare to sham. **(C)** Thrombin activity in EAN rats sciatic nerve compare to sham rats (thrombin activity in sham rats was defined as 100%). Thrombin activity was measured at DPI10, DPI14, and DPI19. Thrombin activity levels were significantly increase in DPI10 in EAN compare to sham (*p* < 0.0001). **(D)** Increased thrombin activity in sciatic nerves of EAN rats in DPI10 is inhibited *in-vitro* by NAPAP and TLCK (thrombin activity in sham rats treated with NAPAP was defined as 100%). Results are presented as mean±SEM. ^**^p ≤ 0.001. Total number of animals in each group: A.B. CS and RR: EAN-24, sham-9. C. Thrombin activity EAN: DPI10-3, DPI14-4, DPI19-4. Sham: DPI10-3, DPI14-5, DPI19-4. D. Thrombin activity *in-vitro* EAN and sham: No treatment-3, NAPAP-3, TLCK-3. Statistical analysis used: Statistical analysis of the differences between clinical scores and rotarod progression were assessed by analysis of variance (ANOVA) with repeated measures. Sidak correction was done for multiple comparisons. The difference in thrombin activity was calculated using two-way ANOVA with Dunnett's correction for multiple comparisons.

EAN rats showed first clinical signs at DPI12 (Figure 1A), including flaccid tail and slight paresis rapidly progressing to severe paraparesis. The mean clinical score of EAN rats was significantly elevated when comparing to the sham group [*F*_(13, 403)_ = 28.08, *p* <0.001 the interaction between all-time points and the two groups was analyzed by repeated measure ANOVA]. Multiple comparisons with Sidak *post-hoc* analysis indicated that the significant increase in clinical score began starting at DPI14, and continued throughout the experiment (2.46 ± 0.35, 0.00 ± 0, mean ± SEM, compared to sham, *p* < 0.01). The mean rotarod walking time of the EAN rats was found to be significantly decreased when comparing to sham group [*F*_(12, 403)_ = 11.87, *p* < 0.001, the interaction between all-time points and the two groups was analyzed by repeated measure ANOVA]. No neurological signs were observed in the sham group during the experiment. EAN rats demonstrated decreased mean rotarod walking times indicated by multiple comparisons with Sidak *post-hoc* analysis beginning at DPI14 (Figure 1B, 41.42 ± 4.8 seconds, mean ± SEM, compared to sham rats, *p* = 0.013).

Thrombin activity in EAN rat sciatic nerves was found to be increased early in the clinical course of the disease, but was normalized in the progression and at the peak of disease. Thrombin activity was significantly elevated in the EAN rat sciatic nerves compared to sham rats at DPI10 prior to the appearance of clinical signs (Figure 1C, 182 ± 17.8 vs. 100 ± 4.85, percent of sham, *p* ≤ 0.005). Thrombin levels measured in the sciatic nerves from EAN and sham rats ceased to be statistically different at DPI14.

Both the highly specific thrombin inhibitor NAPAP and the non-selective thrombin inhibitor TLCK reduced the *in-vitro* EAN excess thrombin activity to sham baseline (Figure 1D, 182.4 ± 17, 120.7 ± 16.5, 107.4 ± 10.7, percent of sham, respectively, *p* < 0.0001 for both inhibitors). Measurements indicate the specificity of this early thrombin activity.

### Inhibition of Thrombin Activity as a Treatment for EAN

We further verified the importance of thrombin-like protease activity in the EAN nerves by performing therapeutic experiments utilizing exogenous protease inhibitors. These included the highly specific thrombin inhibitor NAPAP and the non-selective thrombin and trypsin-like serine protease inhibitor TLCK. The effects of these treatments were compared to saline treated EAN controls.

### Preliminary Treatment Protocols Study

**Figure 2:**
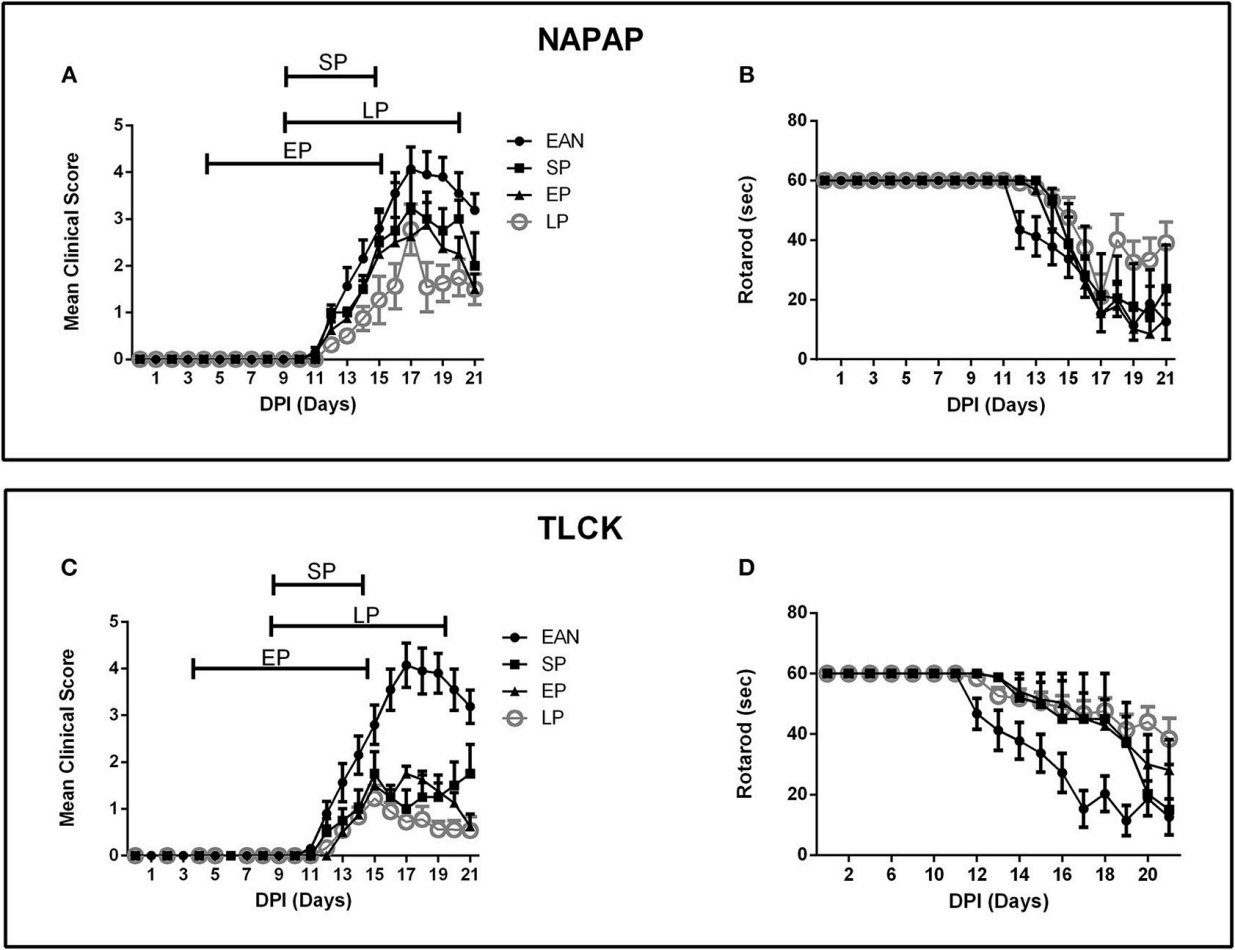
Time course of clinical parameters in EAN rats under different treatment protocols. Comparison between 3 treatment protocols for NAPAP and TLCK in order to evaluate the most effective time frame for treatment. Treatment protocols included early protocol (EP), with daily injections at DPI5-15, late protocol (LP) with daily injections at DPI10-20, and short protocol (SP) with daily injections at DPI10-15. Each protocol was applied in both NAPAP and TLCK. Days are marked DPI following immunization day (DPI0). The bars above mean clinical score graphs mark the length of each treatment protocol. **(A)** Mean clinical scores (CS) with different NAPAP treatment protocols. **(B)** Rotarod (RR) scores with different NAPAP treatment protocols. **(C)** Mean CS with different TLCK treatment protocols. **(D)** RR scores with different TLCK treatment protocols. The late treatment protocol marked with gray circles, was the most effective. Results are presented as Mean±SEM. Total number of animals in each group: For NAPAP (CS, RR): SP-4, LP-18, EP-8, EAN-20. For TLCK (CS, RR): SP-4, LP-16, EP-8, EAN-20. Statistical analysis used: Two-way ANOVA with Tukey test for multiple comparisons was performed for the effect of EAN and progression in time on clinical score and rotarod.

Three protocols were assessed as described in detail in the Methods section: an early pre-clinical protocol (EP), a short protocol (SP) covering the period of disease progression and a long protocol (LP) covering both the early clinical stage and peak of disease (Figure 2). Clinical score and rotarod scores were recorded for all TLCK and NAPAP treatment protocols. Animals were assigned to different protocol groups prior to the development of disease, but later all of them showed clinical signs of EAN.

The effect of NAPAP treatment protocols on clinical score are presented in Figure 2A. Following the onset of clinical deterioration, all treatment protocols were found to significantly reduced disease severity compared to EAN [*F*_(33, 484)_ = 3.38, *p* < 0.001, for the interaction between all-time points and groups, analyses using two-way ANOVA with Dunnett's correction for multiple comparisons]. At disease peak (DPI18) all NAPAP treatment protocols had a similar modest non-significant effect on clinical score relative to EAN [*F*_(3, 31)_ = 1.79, *p* = 0.17, for the comparison between different treatment groups to the EAN, using one-way ANOVA, with Dunnetts' correction for multiple comparisons]. EP and LP treatment protocols improved clinical score during the recovery phase of EAN [*F*_(9, 105)_ = 2.149, *p* = 0.0316 for the interaction between groups and days of recovery calculated using two-way ANOVA with Tukey's *post-hoc* analysis revealing the EP and LP effect]. Motor function assessed by rotarod score detected similar effects of NAPAP treatment. The treatment was not found to improve the rotarod function over the entire course of disease [*F*_(27, 387)_ = 1.29, *p* = 0.16, for interaction between all-time points and groups, Figure 2B].

Following the onset of clinical score deterioration (DPI14) all TLCK treatment protocols were found to be better than EAN [*F*_(39, 598)_ = 11.72, *p* < 0.01, the interaction between all-time points and the groups was analyzed by repeated measure ANOVA, with Dunnett's correction for all treatments compare to EAN]. At disease peak, (DPI17) all treatments improve clinical score [1 ± 0.4, 1.75 ± 0.16, 0.72 ± 0.13, 4.07 ± 1.47, SP, EP, LP, vs. EAN respectively, mean ± SEM, *F*_(3, 40)_ = 25.04, *p* < 0.001] and there was no significant difference between treatment protocols. During the recovery phase (DPI18-20) EP and LP treatments improved clinical score [*F*_(3, 37)_ = 11.57, *p* < 0.001, analyzed by repeated measure ANOVA, followed by Tukey's *post-hoc* analysis, Figure 2C]. Motor function assessed by rotarod score detected similar effects of TLCK treatment. From the beginning of motor deterioration, all treatments had a beneficial effect [*F*_(36, 552)_ = 2.9, *p* < 0.001 for the interaction between all-time points and groups, Figure 2D]. LP treatment was found to enhance recovery compare to EAN [*F*_(3, 37)_ = 3.82, *p* = 0.017 analyzed by repeated measure ANOVA with Tukey's correction].

### Electrophysiology

**Figure 3:**
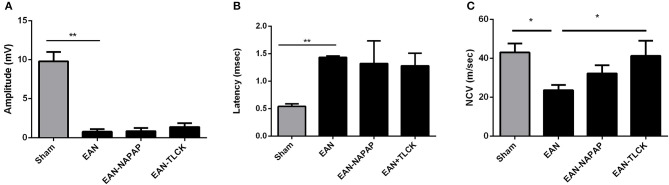
Electrophysiological findings in sham, EAN, and treated EAN, DPI26. Electrophysiological measurements were conducted on the tail nerve. Amplitudes of distal compound muscle action potential (CMAP), distal latency and nerve conduction velocities were evaluated. **(A)** CMAP in sham, EAN, and different treatment protocols. Amplitude was significantly lower in EAN compared to sham rats. **(B)** Distal latency in sham, EAN, and different treatment protocols. Latency was significantly longer in EAN rats. **(C)** Nerve conduction velocities (NCV) in sham, EAN, and different treatment protocols. Velocity was significantly slower in EAN compare to sham rats. This was partially corrected by TLCK treatment. Results are presented as Mean±SEM. ^*^p ≤ 0.05, ^**^p ≤ 0.001. Number of animals in each group (amplitude, latency, NCV): Sham-3, EAN-3, EAN-NAPAP-3, EAN-TLCK-3. Statistical analysis used: One-way ANOVA with Dunnett's correction for multiple comparisons was used to compare the means of electrophysiological measures.

NCV studies were carried out at a late phase of disease (DPI26) since there is a known 1–2 weeks delay between the disease onset and the appearance of electrophysiological changes in both EAN and GBS. Furthermore, performance of electrophysiology studies requires anesthesia, which by morbidity and mortality may have deleteriously affected the clinical measures. All EAN rats demonstrated severe pathology in electrophysiology parameters (Figure 3). Distal amplitude was significantly lower in EAN compared to sham rats (0.76 ± 0.34, 9.8 ± 1.2 mV, respectively, *p* < 0.0001, Figure 3A) and was not significantly improved with either treatment. Latency was longer in EAN compared to sham (1.43 ± 0.02, 0.54 ± 0.03 msec, *p* < 0.0001, Figure 3B), with some improvement with TLCK and NAPAP treatments (no statistical significance, *p* = 0.27, *p* = 0.4, respectively). The results obtained confirm a severe axonal and demyelinating neuropathy in the EAN animals compared to sham. The effects of NAPAP and TLCK treatments on the axonal component of the disease were minimal, as expressed by muscle response amplitude. Nerve conduction velocities were slower in EAN compared to sham (23.6 ± 2.16, 43 ± 3.7 m/s. Figure 3C, *p* = 0.0214) and were significantly improved by TLCK treatment (23.6 ± 2.65, 41.27 ± 7.7 m/s, *p* = 0.047). Significant beneficial effects were demonstrated in the demyelination measure of nerve conduction velocity by TLCK, which is in-line with the more striking effect of TLCK on the clinical measures.

### Immune-Histochemical Evaluation and Splenocyte Proliferation

**Figure 4:**
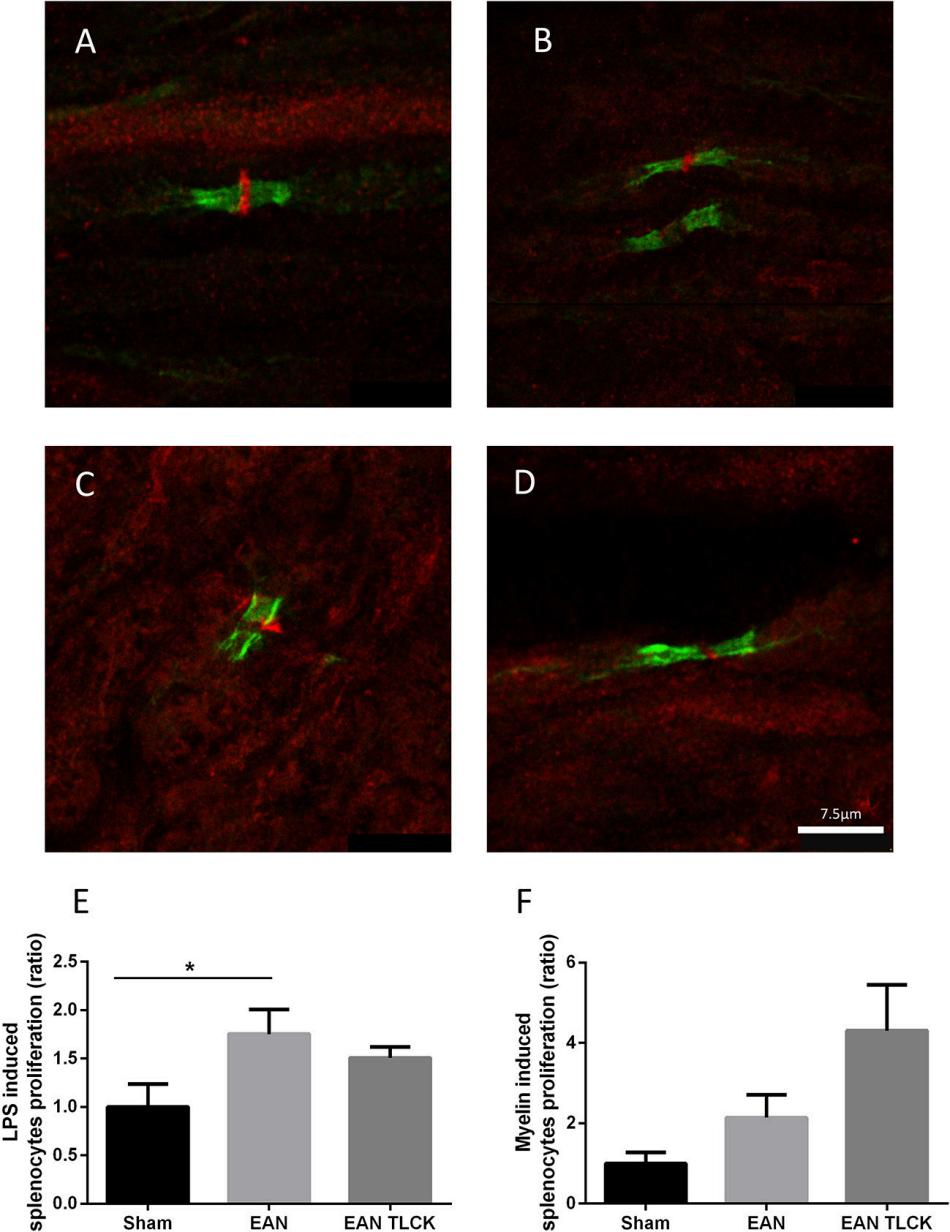
Immune-histochemical staining of nodes of Ranvier and splenocytes proliferation assay. Representative images from EAN, sham and treated rats, taken at DPI32. Normal node architecture was defined as the presence of paranodal CasprI stain, and staining of PAR1 in the node between the paranodal CasprI stains. Sciatic nerve fibers of sham **(A)** EAN **(B)**, EAN treated with NAPAP **(C)** and EAN treated with TLCK **(D)** rats. Nodes of Ranvier taken from EAN showed abnormal lack of PAR1 stain compare to sham rats. TLCK treatment significantly improves the appearance of the nodes. Splenocytes proliferation reaction in response to LPS **(E)** and myelin **(F)** in sham, EAN and EAN treated TLCK rats. Proliferation in EAN in response to LPS was not significantly inhibited by TLCK. Results are presented as Mean±SEM. ^*^p < 0.05. The sciatic nerves have been double-stained for PAR1 (red) and CasprI (green). Scale bar: 7.5 μm. Magnification X630. Immune-histochemistry: four animals were taken from each group (sham, EAN, EAN treated with NAPAP and EAN treated with TLCK), and 7–8 nodes of Ranvier were taken from each animal, to a total number of 30 nodes per group. Proliferation assay: four specimens were analyzed in each group. Experiments were performed in triplicate. Analysis of immune-histochemistry was done using Fisher's exact test. Analysis of proliferation assay was done using one-way ANOVA with Tukey *post-hoc* analysis.

Nerve sections taken from sham, EAN, EAN treated with NAPAP and TLCK were quantitatively analyzed using randomly mixed images. EAN nerve sections showed abnormal structure defined as lack of PAR1 staining as described earlier, in 25 out of 30 images compare to 1 out of 30 in sham sections (*p* = 0.0001, Fisher's exact test, Figures 4A,B). Sections taken from NAPAP and TLCK treated EAN showed abnormal structure in 21 out of 30 nodes and 17 out of 30 nodes (*p* = 0.18, *p* = 0.02 respectively, when compared to EAN, Figures 4C,D).

The beneficial effects of inhibiting thrombin on EAN neuropathy may be due to local inhibition of thrombin in the nerve or through a more general immune-suppressive effect. We tested this possibility by isolating immune cells from the spleens of EAN animal treated by TLCK or sham. The general B-cell and macrophage stimulator LPS induced splenocytes proliferation reaction increase in EAN compare to sham (*p* = 0.01, Figure 4E). TLCK did not affect this proliferation significantly. The specific antigen used to induce EAN in this mode, bovine spinal root myelin induced a small increase in splenocytes proliferation in EAN rats (*p* = 0.37, sham vs. EAN), and the effect of TLCK was to further increase proliferation (*p* = 0.2, sham vs. TLCK treated EAN, Figure 4F).

## Discussion

This study demonstrates specific thrombin and PAR1-related biochemical, histological and electrophysiological changes in peripheral nerves of EAN rats. The initial event is an early elevation of thrombin levels in the sciatic nerve and destruction of the PAR1-associated structures at the node of Ranvier. The functional importance of increased thrombin levels is supported by the major beneficial effect of exogenous thrombin inhibitors on the severity and course of EAN. All treatments in different protocols had various degree of beneficial effect on the clinical and rotatod scores of the EAN rats. This was expressed as a lower disease peak, and as better final outcome. It is interesting to note that EP and SP treatments, which were limited to the time period prior to recovery, had an impact on recovery phase as well. This may indicate a complex series of event induced by early intervention.

The EAN rats developed the expected monophasic diseases course, characterized by a relatively rapid deterioration to paresis, followed by a gradual recovery. The EAN rats showed a significant rise in thrombin activity in the sciatic nerve. The rise in thrombin activity was an early event, noted just prior to the appearance of clinical signs on DPI10. In EAN rats, nerve thrombin activity was higher compared to sham rats and was inhibited to levels close to baseline activity using TLCK, which is a non-selective thrombin and trypsin-like proteases inhibitor. This effect was also found by using the highly specific thrombin inhibitor NAPAP. The significant inhibition caused by NAPAP supports the specific involvement of thrombin rather than other serine proteases in EAN nerve pathology. These results are thus compatible with an early increase in specific thrombin activity which is then probably countered by an increase in endogenous thrombin inhibitors, as can be seen in other reported animal models for neuroinflammation such as the EAE (18, 34).

The peak of measured excess thrombin activity is on day 10 and therefore the treatment protocols included one that preceded this point by 5 days, a short protocol for the period when this elevation occurred and a longer protocol which was initiated from the elevation of thrombin well into the recovery phase. Of these protocols, the long protocol gave significantly better results in the TLCK treatment, thus supporting a role for thrombin-like proteases from the first appearance of clinical signs into the recovery phase. The longer protocol was significantly better in the recovery phase than both other protocols, which ended on day 15. Indeed, following the cessation of treatment on day 15, both protocol groups demonstrated an exacerbation of motor deficit (as measured by rotarod), in contrast to improvement in the long treatment protocol group which were still treated. In the NAPAP group there was an early effect on the severity of disease in all 3 protocols, similar to the TLCK group. The results strongly suggest that at these early time points (DPI10-15) thrombin is specifically important in disease propagation. In contrast, in both the peak of disease and in the recovery phase, the non-selective TLCK treatment was significantly better than NAPAP in all study measures: clinical score, motor deficit, nerve conduction and histology of PAR1 staining at the node of Ranvier. These results suggest that at later stages of disease a more general trypsin and thrombin-like protease activation is important in disease progression. These results suggest that targeting thrombin and trypsin-like proteases that can affect the node of Ranvier through PAR1 is a viable approach to the therapy of GBS.

The node of Ranvier is thought to be a primary structural and functional participant in the pathophysiology of GBS and EAN. The presence of relevant antigens such as gangliosides in the nodes of Ranvier was reported before (38). We have previously found PAR1 localized to the node of Ranvier (28). Thrombin, the main PAR1 activator, was found to cause a sciatic nerve conduction block in a PAR1 mediated manner (28). It is also known that thrombin is related to neuronal inflammation and glial activation (20, 39, 40). In the present study, we found degeneration of the nodes of Ranvier containing PAR1 structure as seen by immune-histochemistry, together with a reduction of nerve conduction velocity. These findings further support the node of Ranvier as an important participant in GBS and EAN pathophysiology. The mode of action of the treatments used in the present study is hypothesized to be essentially protease inhibition and specifically thrombin inhibition since one of the substances used is a highly selective for this protease. Direct evidence is provided to substantiate that the increased thrombin activity is inhibited by both TLCK and NAPAP. The proteolytic consumption of PAR1 is demonstrated in the present results and supports this hypothesis. Thus, the hypothesis that excess activation of thrombin cause first dysfunction through the activation of PAR1, and then tissue destruction at the node of Ranvier, is substantiated by a number of findings in the present study. Further modes of action are certainly possible, and subjected to future research. Thrombin causes a secretion of interleukins from blood dendritic cells (41), it is therefore reasonable to suspect that its mechanism of action in EAN may include additional immunological effects besides its role in the node of Ranvier. In our hands, using splenocytes proliferation assay, TLCK was not found to have a significant immunological effect. There are very few studies on the modulation of immune and inflammatory cells by thrombin inhibition and this may well be systematically approached in further research.

Both node of Ranvier structure and nerve conduction function were partially restored using thrombin inhibitors. Electrophysiological studies demonstrated the expected lower CMAP amplitude as well as slower nerve conduction velocity and longer latency in the EAN rats compared to sham. The treatment with thrombin inhibitors improved these functions achieving statistical significance in measures of nerve conduction velocity. Lack of statistical significance in other electrophysiological measures might be due to the small number of animals in each group. This number of animals (three from each group) was set due to the known high risk of mortality during this part of the experiment and the great importance in continuing monitoring the treatment effect over the study period (electrophysiology was done on DPI26). The statistical significance found in nerve conduction velocities, despite the limited number of animals, may indicates a solid therapeutic effect. Alternatively, since the mechanisms for the changes in electrophysiology remains elusive, amplitude and latency may not participate in its pathophysiology.

Current GBS treatments include IVIG (42), plasma exchange (43) and supportive care. The natural history of GBS is to eventually subside, although only about 57% of the patients show complete cure without treatment. The underlying mechanism of these treatments is partially understood as well of the disease pathophysiology itself. Our study suggests a novel treatment target not previously used for treatment of GBS, and improves our understanding of GBS pathophysiology.

Thrombin inhibitors have anticoagulation properties. Although this might be of use in some patients with the appropriate comorbidities, they carry risks as well. The non-selective thrombin inhibitor TLCK used in this study caused bleeding tendency in laboratory animals (was detected post mortem, not shown). TLCK caused more bleeding than NAPAP, perhaps due to its wide range of activities. Although NAPAP did not cause evident bleeding in this study, it is a potent thrombin inhibitor and the use of similar medication in GBS would require caution. Further research is needed in order to develop more selective thrombin inhibitors, in order to allow separation of the inflammatory and anti-coagulatory effects. The current use of these agents allow better understanding of GBM underlying mechanism, for future development of therapies.

In conclusion, thrombin and PAR1 inhibitors offer new directions in the treatment and understanding of GBS. Further research is needed in order to find more specific thrombin inhibitors and in order to understand the interplay between thrombin and the damage to nodes of Ranvier. Future structural, physiological and immunological evaluation of the changes associated with thrombin inhibition in EAN rats may shed light on the mechanism by which inhibition of PAR1 activation induces its beneficial effect.

## Ethics Statement

This study was carried out in accordance with the recommendations of the animal welfare committee (M-06-004). All experiments were approved by the animal welfare committee (M-06-004) and appropriate measures to avert pain and suffering to the rats were taken.

## Author Contributions

ES-S: planning of experiments, analysis of results, writing manuscript; RA: planning of experiments and execution; CS: execution and analysis of nerve conduction experiments; OG: writing manuscript, consultation regarding electrophysiology; SG: analysis of results, writing manuscript; JC: planning of experiments, analysis and interpretation of results; AD: consultation regarding electrophysiology, interpretation of results. All authors have approved the final manuscript. The raw data supporting the conclusions of this manuscript will be made available by the authors, without undue reservation, to any qualified researcher.

### Conflict of Interest Statement

The authors declare that the research was conducted in the absence of any commercial or financial relationships that could be construed as a potential conflict of interest.
